# Calcium chloride and/or sodium selenite: an effective strategy for extending vase life and alleviating stem bending in gerbera “Stanza” flowers

**DOI:** 10.1186/s12870-026-09163-9

**Published:** 2026-06-10

**Authors:** Samah M. Youssef, Rasha S. El-Serafy

**Affiliations:** 1 Horticulture Department, Faculty of Agriculture, Fayoum University, Fayoum, 63514 Egypt; 2 Horticulture Department, Faculty of Agriculture, Tanta University, Tanta, 31527 Egypt

**Keywords:** Cell wall degrading enzyme, Gerbera, Ion leakage, Lignin, Scape bending, Selenium, Vase life

## Abstract

**Purpose:**

Despite the importance of gerbera in the international market, stem bending is still the biggest problem for gerbera cut flowers during the post-harvest stage, as it reduces the flowers’ vase life and their market value.

**Methods:**

To address this problem, the effects of different vase solutions containing calcium chloride (CaCl_2_) and sodium selenite (Na_2_SeO_3_) on the longevity and stem curvature of gerbera ‘Stanza’ flowers were investigated using repeated-measures ANOVA. The treatments were: distilled water (control, T1), 1 g L^–1^ (T2) and 2 g L^–1^ (T3) of CaCl_2_, 1 mg L^–1^ (T4) and 2 mg L^–1^ (T5) of Na_2_SeO_3_, and 1.5 g L^–1^ CaCl_2_ plus 1.5 mg L^–1^ Na_2_SeO_3_ (T6).

**Results:**

Gerbera cut flowers subjected to T6 of vase solution exhibited a significantly longer vase life (20 days) and a substantially lower degree of stem bending (25.7°). Moreover, these flowers achieved the lowest significant ion leakage (IL) in petals, and an inhibition in the cellulase (CEL), polygalacturonase (PG), xylanase (XYL), and pectinase (PT) activities, along with noticeably greater levels of catalase (CAT) activity and lignin content until the last day of sampling. Automatic linear modeling analysis revealed CEL, PT, PG, lignin content, and XYL were selected as the most contributing attributes in determining the degree of stem bending.

**Conclusions:**

It can be recommended that using calcium and/or selenium can be regarded as an effective substitute vase solution to maintain gerbera flowers and reduce bending stems over the post-harvest period.

## Introduction

Cut flowers longevity and quality are critical economic factors for marketing [1], and their market value decreases as a result of the problems caused by their short vase life and low quality after harvest. Gerbera (*Gerbera jamesonii*), a member of the Asteraceae family [2], is referred to as the Transvaal daisy. According to the global tendencies in floriculture (FloraHolland 2007), it ranks fourth among cut flowers [3]. Its commercial value is often affected by post-harvest problems. This creates a pressing economic and scientific need for effective preservations solutions [4].

Although its stem is leafless, a cut gerbera flower undergoes significant water loss via transpiration directly through stomata on floral-head [5], which is the primary cause of its shortened vase life [6]. Furthermore, stem bending is fundamental problem that severely limits the vase life and commercial attractiveness of many gerbera cultivars [7].

Stem bending connected with low levels of stem lignin, and is linked to the absence of a thick cylinder of sclerenchyma located 20–30 cm below the capitulum [8]. Bacteria within the vase solution may be the most frequent source of xylem obstruction that affects water uptake [7], but bacteria can also harm flower scape structure and accelerate senescence by producing harmful compounds [4]. Furthermore, one of the primary factors influencing stem bending, and consequently, the longevity and quality of gerbera cut flowers, is their ability to absorb and retain water [9].

Several researches on improving of the postharvest properties of cut flowers have been published, but only a few researches address the problem of stem bending in gerbera. The use of vase solutions containing stimulants capable of strengthening cut gerbera stems has become increasingly important. This has enhanced the benefits of their use in post-harvest technologies in the flower industry.

Calcium (Ca) is an essential regulator of the growth and development of plants, and is absorbed as a cation that is divalent [10]. Furthermore, it contributes to the development of cell walls and membranes by producing calcium pectates in the middle-lamella [11], and inhibits the activity of the polygalacturonase-enzyme during division of cells [12]. Pectin is an important structural polysaccharide, which keeps the cell wall’s hardness maintained [13]. Ca ions maintain the rigidity and firmness of the cell wall by catalyzing the cross-linking of pectin polymers to form cell wall networks [14]. The buildup of Ca makes the cell wall’s mechanical strength more powerful [15], which gives plants mechanical support. Moreover, Ca blocks stomatal opening and decreases transpiration [16].

Selenium (Se) offers suitable features to be a cost-effective and eco-friendly alternative to formulating flower preservative solutions to inhibit ethylene production [17]. It is an essential trace element that regulates plant growth and activates defense mechanisms that scavenge ROS in response to various biotic and abiotic stresses [18]. The primary biological activities of Se are attributed to seleno-proteins, which include this element as a component of the amino acids seleno-cysteine (SeCys) and seleno-methionine (SeMet), in addition as the antioxidants substances, enzyme co-factors [19], lipids, and specialized metabolites. Lu et al. [20] revealed that Se could control the water balance of the plants and lessen oxidative damage throughout the postharvest aging process by adjusting the osmotic pressure of cut *Lilium longiflorum* flowers, improving the plants’ postharvest performance. Lu et al. [21] reported that postharvest treatment with selenium of *Eustoma grandiflorum* flowers postponed senescence and markedly increased water uptake by enhancing antioxidant activity and lowering ethylene production.

Se could prolong the vase life of cut flowers snapdragon (*Antirrhinum majus*), lily (*Lilium longiflorum*), and lisianthus (*Eustoma grandiflorum*), according to reports by Tognon et al. [22], Lu et al. [20] and Lu et al. [21], respectively. However, the effect of Se either alone or combined with Ca on the stem bending or vase life of cut gerbera flowers has not yet been reported. Hence, it will be intriguing to discover how Se influences the quality and longevity of cut gerbera flowers, which will provide further information about its application in the cut floral industry. Therefore, the purpose of this investigation was to assess the positive effects of CaCl_2_ and/or Na_2_SeO_3_ on improving the postharvest quality of gerbera cut flowers by prolonging vase life, and to elucidate the physiological, biochemical and cell-degrading enzyme mechanisms underlying stem bending reduction.

## Materials and methods

### Cut flower materials and preparation

Gerbera (*Gerbera jamesonii* cv. Stanza) flowers were purchased from a commercial grower and brought directly to the laboratory of Horticulture Department, Faculty of Agriculture, Fayoum University. The stage of harvesting was when the two outer whorls of flowers in the floral head displayed mature stamens. The stems were cut to 50 cm in length, and the stem’s bending angle was measured (15^◦^ with respect to vertical position). The stems of gerbera were re-cut under deionized water for preventing air bubbles, following cutting, a central cavity was consistently seen the base of the inflorescence. The experiment was conducted twice in the periods of 1st December 2024 and 1st January 2025.

### Vase solution treatments

Gerbera cut flowers were subjected to different vase solutions as follows: distilled water as a control (T1), 1 g L^–1^ (T2) and 2 g L^–1^ (T3) of CaCl_2_, 1 mg L^–1^ (T4) and 2 mg L^–1^ (T5) of Na_2_SeO_3_, and 1.5 g L^–1^ CaCl_2_ plus 1.5 mg L^–1^ Na_2_SeO_3_ (T6). Each individual cut flower was placed in glass vases containing 250 mL vase solution. The preservative solutions were supplemented with 20 g L^–1^ sucrose. Cut flowers were placed at room temperature (20 ± 3^°^C and 18 ± 3^°^C for the two experiments, respectively), with 16 /8 h lightness/darkness (cold white-fluorescent lamps) at a light intensity of 15 µmol m^− 2^ s^− 1^ radiation, and 65 ± 5% of relative humidity. Each treatment was replicated 5 times, and 5 cut flower stems were used for each treatment within each replicate. All the characters of cut flowers were assessed every three days starting from the 3rd to 12th days of vase life.

### Vase life and total solution uptake (TSU)

Cut flowers were checked daily for longevity assessment and the vase life was terminated when the flowers lost their decorative value e.g. loose turgidity, 75% of the ray floret fading or wilting, stem bending under the flower head, or floret abscission, were considered indicators of vase life termination [23]. Total solution uptake (TSU in mL) was estimated as the variance between the volumes of solution at the offing and end of the treatment during the vase life [24].

### Relative Fresh Weight (RFW)

The RFW (%) of gerbera cut flowers was calculated using the formula:

RFW (%) = $\:\frac{Wt}{W0}\times\:100$, where Wt is the weight of the flower stem (g) at t =days 3, 6, 9, 12, and W0 is the weight of the same flower stem at day 0 [25].

### Flower Diameter (FD)

Flower head diameter (cm) was estimated at days of 3, 6, 9, 12 of the vase life experiment using a digital caliper (Vernier Accuracy, China).

### Stem Bending (SB)

At the beginning of the experiment, stems were placed in vases at a 15^°^ inclination to the vertical. Stem bending was assessed by analyzing the associated change in the placement of the floral head. The angle between the main stem and the stem directly below the flower head was calculated using a protractor. Gerbera-cut flowers were assessed for flower stem bending on a scale of 0 to 4 as follows: 0 = for scape-bending to 15^°^, 1 = for scape-bending between 15^°^ and 25^°^, 2 = for scape-bending between 25^°^ and 65^°^, 3 = for scape-bending between 65^°^ and 90^°^, and 4 = for flowers scape-bending more than 90^°^ [26].

### Membrane Stability Index (MSI)

Samples from the second outer circumference of petals were used for estimating MSI (%) conforming to the procedure outlined by Almeselmani et al. [27].

### Total Anthocyanin Content (TAC)

The anthocyanin content (mg g^− 1^ FW) of gerbera-ray florets was determined based on the method of Giusti and Wrolstad [28].

### Total Phenols Compounds (TPC)

Total phenols (mg GAE g^− 1^ FW) estimation was done spectrophotometrically (Shimadzu Company, Kyoto, Japan) using samples collected from the second outer circumference of petals using Folin–Ciocalteu’s reagent in accordance with the method outlined by Shin et al. [29].

### Ion Leakage (IL)

Applying the following formula IL (%) = $\:\frac{EC1}{EC2}\times\:100$, the ion leakage (IL) of petal was calculated [30], since EC1 and EC2 are the electric conductivity before and after placing the samples in a hot water bath at 90^°^ C, respectively.

### Malondialdehyde Determination (MDA)

MDA (µmol^− 1^ g FW) content was used to terminate the amount of lipid peroxidation, in accordance with Hodges et al. [31] procedures.

### Hydrogen peroxide evaluation (H_2_O_2_)

Gerbera petals’ H_2_O_2_ (µmol^− 1^ g FW) content was measured using the technique of Liu et al. [32].

### Cell-wall-degrading-enzymes and catalase activities

The enzymes of cellulase (CEL), polygalacturonase (PG), and xylanase (XLN) were determined following the methodology of Miller [33]. Pectinase (PT) activity was calculated using the extraction method of Payasi and Sanwal [34], in accordance with Collmer et al. [35]. Catalase (CAT) activity was measured using the method proposed by Chandlee and Scandalios [36]. Enzyme activity was expressed as Unit mg^− 1^ Protein in the fresh weight of cut stem segment.

### Lignin content

Lignin content (%) at the stem sections was estimated on the last day of the vase period for all treatments. Lignin content was ascertained using the method described by A.O.A.C [37].

### Statistical analysis

Prior to pooling, Levene’s test for homogeneity of variance was applied across both experimental cycles for all measured parameters. As no significant variance heterogeneity was detected between cycles (*p* > 0.05), data from both cycles were deemed suitable for combined analysis, as also recommended by Dewey and Lu [38]. The pooled data were then analyzed employing repeated-measures-ANOVA. The data was statistically analyzed using IBM SPSS-AMOS 24 [39], assuming that different sampling times (vase day) as dependent factor, and treatments (preservative solutions) were regarded as independent variables. The data were firstly subjected for Shapiro–Wilk (normality test) and Mauchly’s sphericity test. However, a randomized complete design by Analysis of Variance (ANOVA) was used to determine the vase life, total solution uptake, and lignin content. Subsequently, Tukey test was used to calculate mean separations with a significance level of 0.01. Results were presented using mean values ± standard error (SE). Automatic linear modeling (ALM) was done using the statistical software IBM SPSS.

## Results

### Vase life and total solution uptake

**Fig. 1:**
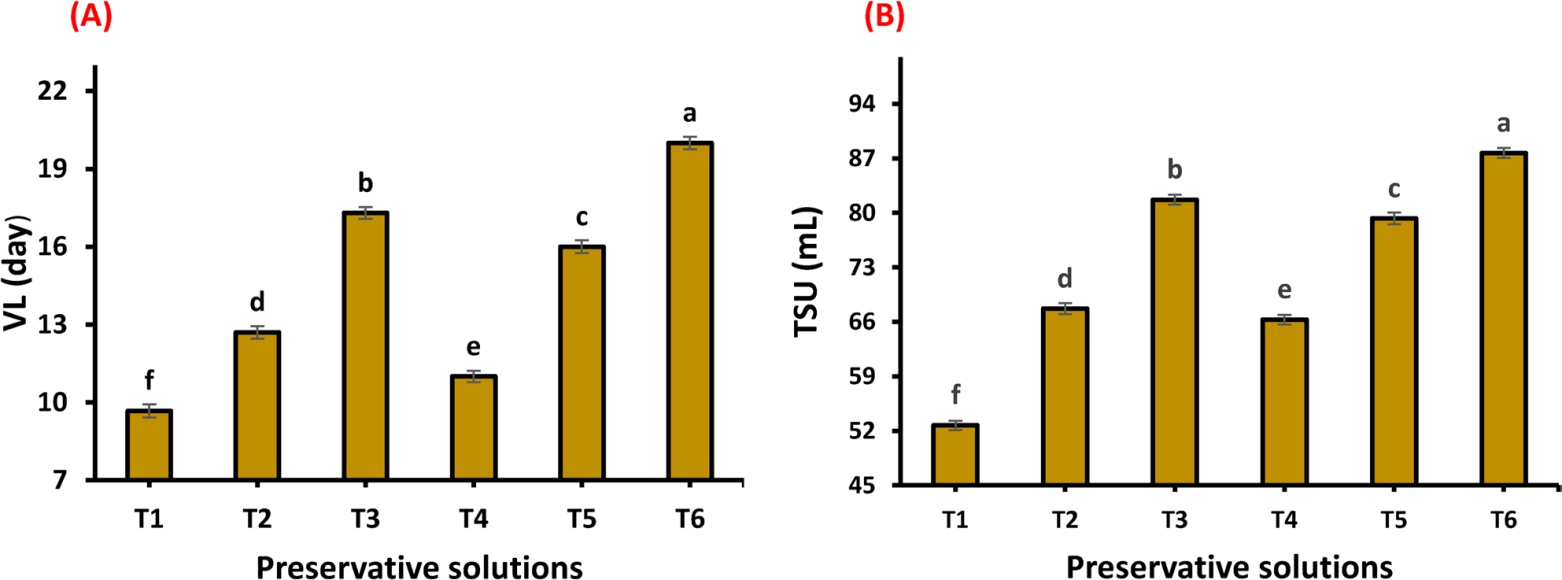
Vase life (VL; **A**), and total solution uptake (TSU; **B**) of *Gerbera jamesonii* “Stanza” cut flowers affected by varied preservative solutions of distilled water as a control (T1), 1 g L^–1^ (T2) and 2 g L^–1^ (T3) of CaCl_2_, 1 mg L^–1^ (T4) and 2 mg L^–1^ (T5) of Na_2_SeO_3_, and combined 1.5 g L^–1^ CaCl_2_ with 1.5 mg L^–1^ Na_2_SeO_3_ (T6) over different sampling times. Bars represent mean values ± standard error (SE). Not identical letters signify a substantial difference between the mean values as per Tukey test (*p* ≤ 0.01)

CaCl_2_ combined with or without Na_2_SeO_3_ effectively extended the longevity and increased the TSU of the cut gerberas as compared with the control flowers (Fig. 1A and B). The flowers with the longest vase life and the maximum solution uptake were those exposed to T6 vase solution. As the vase life of these cut flowers increased by 106.8% as compared with control flowers (Fig. 1A), and TSU has increased to 87.7 mL flower^− 1^ during the vase life, which recorded 66.4% higher than control flowers (Fig. 1B). T3 solution ranked second in this respect followed by T5 solution.

### Relative fresh weight

**Fig. 2:**
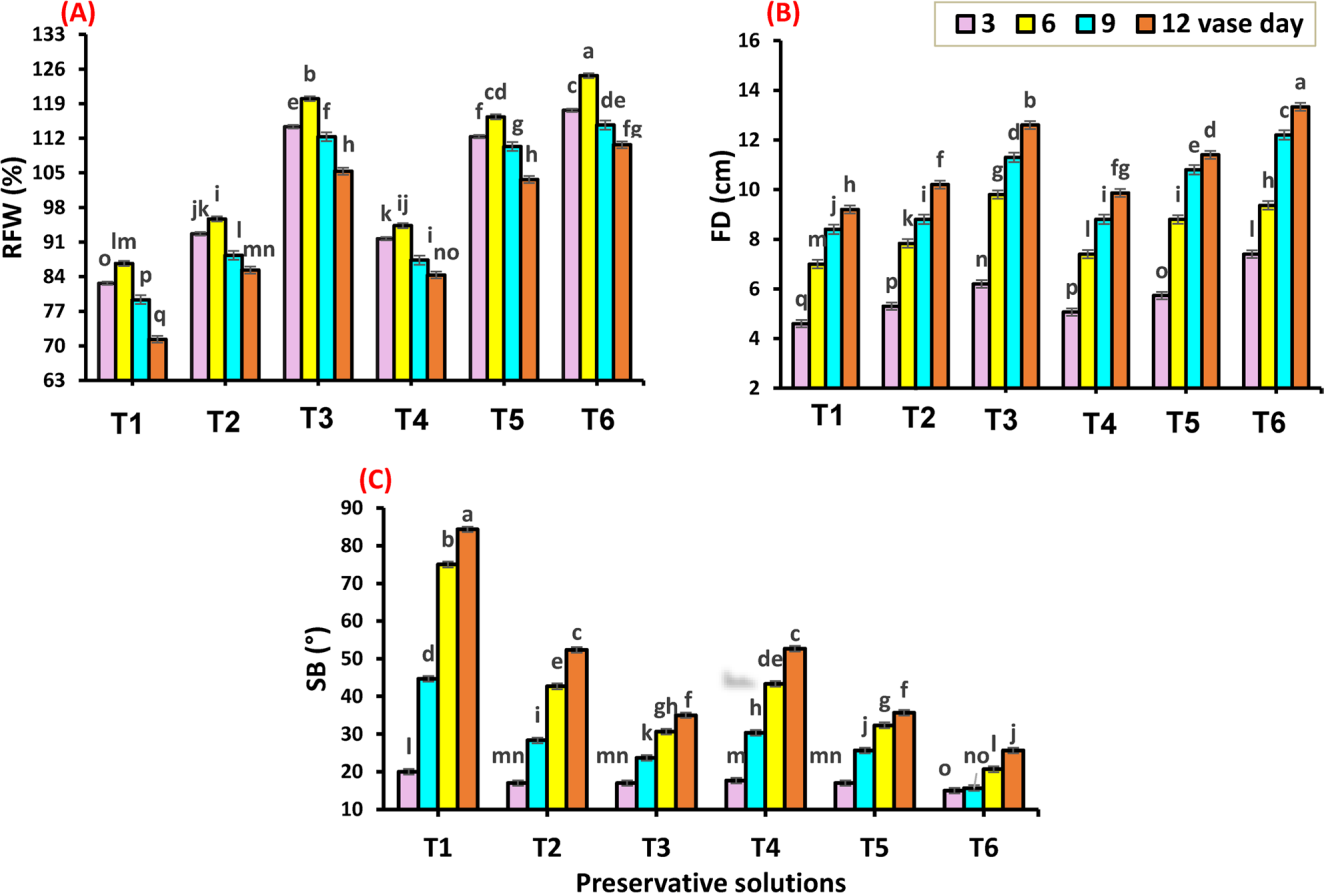
Relative fresh weight (RFW; **A**), flower diameter (FD; **B**), and stem bending (SB; **C**) of *Gerbera jamesonii* “Stanza” cut flowers affected by varied preservative solutions of distilled water as a control (T1), 1 g L^–1^ (T2) and 2 g L^–1^ (T3) of CaCl_2_, 1 mg L^–1^ (T4) and 2 mg L^–1^ (T5) of Na_2_SeO_3_, and combined 1.5 g L^–1^ CaCl_2_ with 1.5 mg L^–1^ Na_2_SeO_3_ (T6) over different sampling times. Bars represent mean values ± standard error (SE). Not identical letters signify a substantial difference between the mean values as per Tukey test (*p* ≤ 0.01)

The flowers treated with CaCl_2_ or Na_2_SeO_3_ had a higher RFW retention, especially for the higher doses (Table 1). Specifically, the RFW of flowers treated with T6 was 46% higher than that of the control flowers. The RFW of gerbera cut flowers increased as the post-harvest period was extended from 3 to 6 days, subsequently steadily decreased (Table 1). Furthermore, the lowest RFW was recorded after 12 days of storage, which was 8.34% lower than on day 3 (from 101.9 to 93.4). Flowers supplemented with CaCl_2_ or/and Na_2_SeO_3_ were able to maintain a higher RFW than untreated-flowers at 3, 6, and 9 days of storage before starting to decline at day 12, although still higher than the untreated-flowers (Fig. 2A).

### Flower diameter

A gradual increase in flower diameter was observed for all preservative solutions used including the control (Table 1). Treated flowers subjected to T6 solution showed the maximum flower diameter increase (from 7.30 to 10.6 cm), which presented 45% higher than the diameter of control flowers. Considering the comparison of different sampling times, the flower diameter of cut flowers expanded progressively with the extension of the vase life period, and the highest petal expansion was measured on day 12 of experiment at a rate of 94% over that of day 3. Over the period of 12 days of experiment, the flower diameter of both treated and control flowers expanded noticeably (Fig. 2B).

**Table 1:** Relative fresh weight (RFW), flower diameter (FD), and stem bending (SB) in gerbera cut flowers affected by varied preservative solutions and different sampling times (vase day)

Treatments	RFW	FD	SB
	(%)	(cm)	(^°^)
**Preservative solutions**			
Distilled water (control; T1)	80.0 ± 0.19f	7.30 ± 0.20f	56.0 ± 0.16a
1 g L^− 1^ CaCl_2_ (T2)	90.5 ± 0.18d	8.03 ± 0.21d	35.1 ± 0.16c
2 g L^− 1^ CaCl_2_ (T3)	113.0 ± 0.16b	9.98 ± 0.20b	26.6 ± 0.17e
1 mg L^− 1^ Na_2_SeO_3_ (T4)	89.4 ± 0.19e	7.78 ± 0.22e	36.0 ± 0.16b
2 mg L^− 1^ Na_2_SeO_3_ (T5)	110.7 ± 0.20c	9.18 ± 0.21c	27.7 ± 0.17d
1.5 g L^− 1^ CaCl_2_ plus 1.5 mg L^− 1^ Na_2_SeO_3_ (T6)	116.9 ± 0.17a	10.6 ± 0.22a	19.3 ± 0.16f
**Different sampling times (vase day)**			
3rd	101.9 ± 0.14b	5.72 ± 0.19d	17.3 ± 0.10d
6th	106.3 ± 0.15a	8.37 ± 0.19c	28.1 ± 0.14c
9th	98.7 ± 0.13c	10.1 ± 0.19b	40.8 ± 0.12b
12th	93.4 ± 0.14d	11.1 ± 0.19a	47.6 ± 0.12a

Values represent means ± standard error *SE*. Not identical letters signify a substantial difference between the mean values as per Tukey test (*p* ≤ 0.01)

### Stem bending

The results exhibit a negative correlation between vase solutions and stem bending of gerbera cut flowers (Table 1). Notably, the greatest protection of the stem bent was observed with T6 solution, resulting in a 65.6% decrease relative to the control flowers. Additionally, the same table reveals the positive correlation between the degree of stem bending and different sampling times, as the highest stem bending (47.6^**°**^) was given when the vase life period was extended to 12 days. All flowers under study displayed no symptoms of stem bending on the first day 3 of sampling (Fig. 2C), while the maximum degree of stem bending was noticed on day 12 then 9 days of vase life, which recorded 84.3^**°**^ and 75^**°**^ in the stem of control flowers, respectively. Surprisingly, no discernible degree of bending in the stems of the flowers treated with T6 up to day 12. The bending degree was 25.7^**°**^, and it is considered the second degree bending according to the classification referred to in Sect. 2.6. Similarly, compared to control flowers, high concentrations of CaCl_2_ or Na_2_SeO_3_ dramatically decreased stem bending until day 12. This indicates that the applied preservation solutions significantly affected the straightness of the gerbera flower stem.

### Membrane stability index

**Fig. 3:**
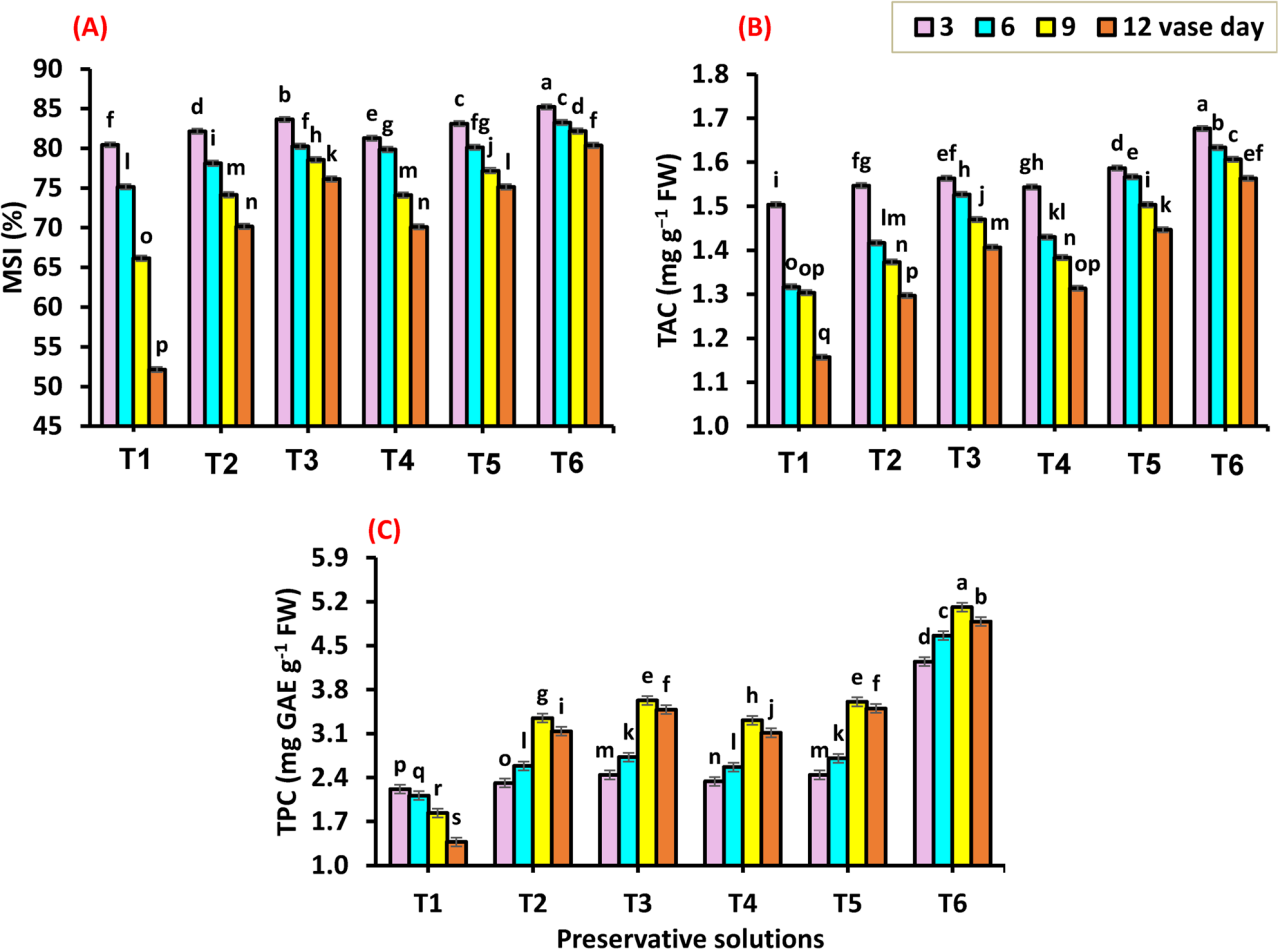
Membrane stability index (MSI; **A**), total anthocyanin content (TAC; **B**), and total phenols content (TPC; **C**) levels of *Gerbera jamesonii* “Stanza” cut flowers affected by varied preservative solutions of distilled water as a control (T1), 1 g L^–1^ (T2) and 2 g L^–1^ (T3) of CaCl_2_, 1 mg L^–1^ (T4) and 2 mg L^–1^ (T5) of Na_2_SeO_3_, and combined 1.5 g L^–1^ CaCl_2_ with 1.5 mg L^–1^ Na_2_SeO_3_ (T6) over different sampling times. Bars represent mean values ± standard error (SE). Not identical letters signify a substantial difference between the mean values as per Tukey test (*p* ≤ 0.01)

Membrane deterioration was considerably decreased under CaCl_2_ and/or Na_2_SeO_3_ applications in comparison to control flowers, which rapidly lost their membrane permeability (Table 2). T6 solution had the highest MSI as increased by 21% over the control flowers. Results presented in the same table show general trend of diminishing MSI throughout the vase period, with the highest membrane deterioration observed in 12 days (70.7%). All CaCl_2_ or Na_2_SeO_3_ treatments overcame these adverse consequences and sustained the MSI higher than the control flowers for up to 12 days (Fig. 3A). Likewise, on day 12, flowers treated with T6 solution exhibited 54% greater in the membrane integrity relative to control flowers (from 52.2 to 80.4%).

### Total anthocyanin content

The TAC value of all CaCl_2_ or Na_2_SeO_3_ treated flowers was increased in comparison to control flowers (Table 2). The maximum TAC of gerbera-ray florets was observed in T6 treated flowers, which was 22.7-fold higher than the control. The TAC value was decreased during vase life period, being lowest (1.36 mg g^− 1^ FW), at the last day of sampling (day 12). CaCl_2_ and/or Na_2_SeO_3_-treated flowers showed noticeably higher TAC on day 12 of their vase period as compared to control flowers. In addition, the flowers of T6 exhibited higher TAC by about 34.5% by the last day of sampling than control flowers (Fig. 3B).

**Table 2:** Membrane stability index (MSI), total anthocyanin content (TAC), and total phenols content (TPC) in gerbera cut flowers affected by varied preservative solutions and different sampling times (vase day)

Treatments	MSI	TAC	TPC
	(%)	(mg g^− 1^ FW)	(mg GAE g^− 1^ FW)
**Preservative solutions**			
Distilled water (control; T1)	68.5 ± 0.04f	1.32 ± 0.02f	1.89 ± 0.002e
1 g L^− 1^ CaCl_2_ (T2)	76.1 ± 0.03e	1.41 ± 0.03e	2.85 ± 0.002c
2 g L^− 1^ CaCl_2_ (T3)	79.7 ± 0.04b	1.49 ± 0.03c	3.07 ± 0.002b
1 mg L^− 1^ Na_2_SeO_3_ (T4)	76.3 ± 0.05d	1.42 ± 0.02d	2.83 ± 0.002d
2 mg L^− 1^ Na_2_SeO_3_ (T5)	78.9 ± 0.03c	1.53 ± 0.03b	3.06 ± 0.001b
1.5 g L^− 1^ CaCl_2_ plus 1.5 mg L^− 1^ Na_2_SeO_3_ (T6)	82.8 ± 0.04a	1.62 ± 0.02a	4.73 ± 0.002a
**Different sampling times (vase day)**			
3rd	82.6 ± 0.03a	1.57 ± 0.02a	2.67 ± 0.001d
6th	79.5 ± 0.04b	1.48 ± 0.03b	2.89 ± 0.001c
9th	75.4 ± 0.04c	1.44 ± 0.02c	3.47 ± 0.001a
12th	70.7 ± 0.04d	1.36 ± 0.02d	3.25 ± 0.001b

Values represent means ± standard error *SE*. Not identical letters signify a substantial difference between the mean values as per Tukey test (*p* ≤ 0.01)

### Total phenols content

Throughout the vase life evaluation, TPC in flowers subjected to T6 was substantially greater than those in the other vase solutions (Table 2) with a 150% increase than control flowers. However no statistically significant differences were found between CaCl_2_ and Na_2_SeO_3_ at the higher concentration. It is evident that the TPC of petals increased significantly until day 9 of vase life period, after that it began to decrease markedly until the last day of vase life (12th days; Table 2). The TPC climbed swiftly, particularly from the 3rd day to the 9th day, whereas it started to decline thereafter on the 12th day of the vase period for all vase solution treatments (Fig. 3C). Similarly, the T6–flowers produced the highest TPC (5.11 mg GAE g^–1^ FW) up to day 9 of vase life.

### Ion leakage

**Fig. 4:**
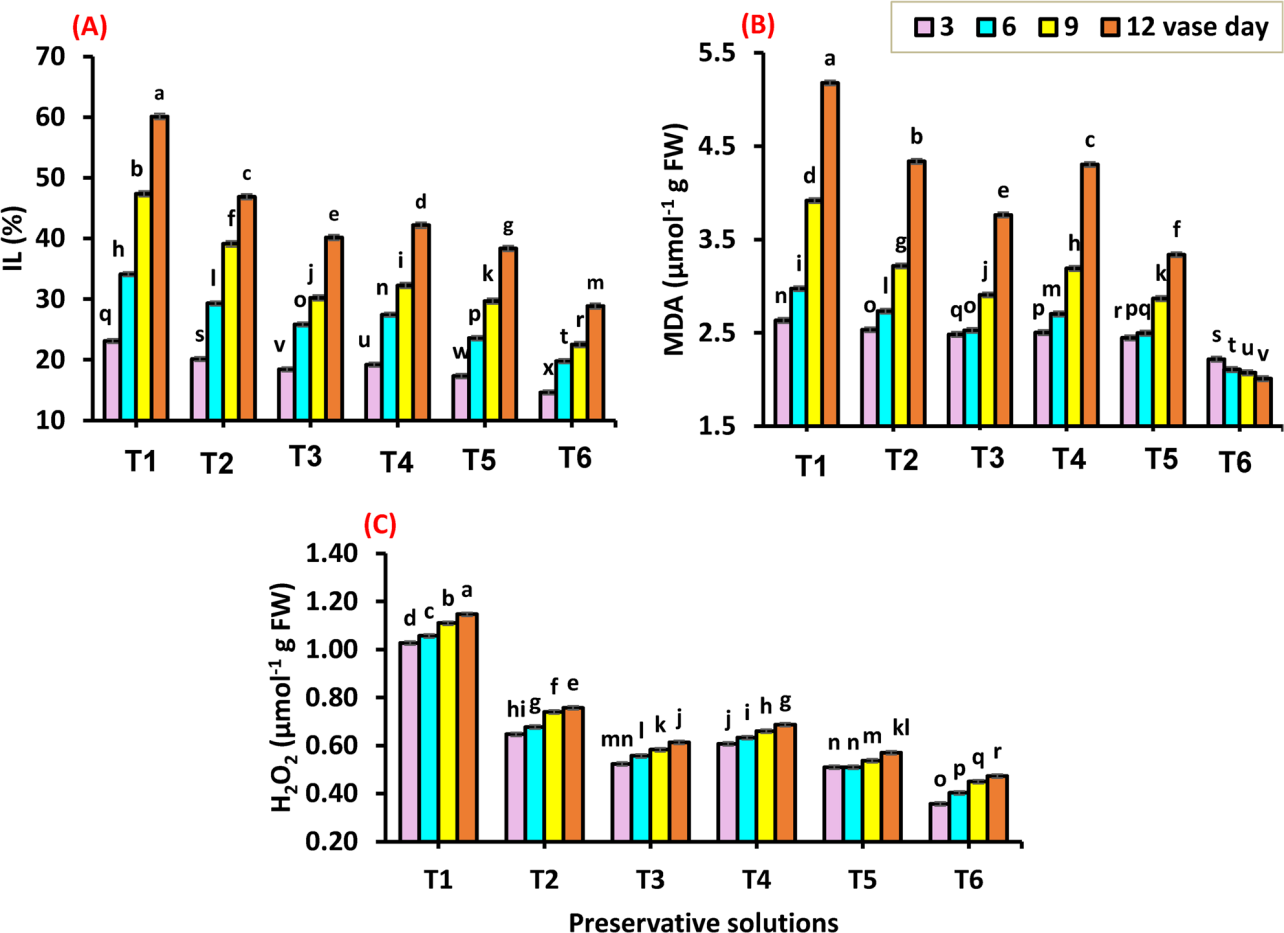
Ion leakage (IL; **A**), malondialdehyde (MDA; **B**), and hydrogen peroxide (H_2_O_2_; **C**) levels of *Gerbera jamesonii* “Stanza” cut flowers affected by varied preservative solutions of distilled water as a control (T1), 1 g L^–1^ (T2) and 2 g L^–1^ (T3) of CaCl_2_, 1 mg L^–1^ (T4) and 2 mg L^–1^ (T5) of Na_2_SeO_3_, and combined 1.5 g L^–1^ CaCl_2_ with 1.5 mg L^–1^ Na_2_SeO_3_ (T6) over different sampling times. Bars represent mean values ± standard error (SE). Not identical letters signify a substantial difference between the mean values as per Tukey test (*p* ≤ 0.01)

Results illustrates that CaCl_2_ and/or Na_2_SeO_3_ at any level decreased the IL of cut flowers, as compared to their respective controls (Table 3). Furthermore, the lowest IL was recorded by T6-flowers as showed a decrease of about 48% as compared to control flowers. The levels of IL in the petals harvested on days 6, 9 and 12 of vase life were higher by 42, 78 and 128%, respectively in comparison to the first day (3rd day) of sampling (Table 3). Data showed that CaCl_2_ and/or Na_2_SeO_3_ decreased IL levels up to the day 12 as compared to the control (Fig. 4A). By day 12, the least IL level (29%) was obtained by T6 treatment, but the maximum level (60%) in the control.

### MDA

In comparison with control flowers, T6- flowers showed a reduction in MDA levels of about 43% (Table 3). The MDA level of cut flowers on day 12 increased by 55% relative to that of day 3 (Table 3). All CaCl_2_ and Na_2_SeO_3_ treatments had a favorable impact on lowering MDA levels throughout vase life period (Fig. 4B). Moreover, on the day 12, the lowest (2.01 µmol^–1^ g FW) and the greatest level of MDA (5.18 µmol^–1^ g FW) were related to T6 and control treatments, respectively.

**Table 3:** Ion leakage (IL), malondialdehyde determination (MDA), and hydrogen peroxide (H_2_O_2_) in gerbera cut flowers affected by varied preservative solutions and different sampling times (vase day)

Treatments	IL	MDA	H_2_O_2_
	(%)	(µmol^− 1^ g FW)	
**Preservative solutions**			
Distilled water (control; T1)	41.2 ± 0.004a	3.68 ± 0.001a	1.09 ± 0.001a
1 g L^− 1^ CaCl_2_ (T2)	33.9 ± 0.003b	3.21 ± 0.001b	0.71 ± 0.001b
2 g L^− 1^ CaCl_2_ (T3)	28.7 ± 0.004d	2.92 ± 0.001d	0.57 ± 0.001d
1 mg L^− 1^ Na_2_SeO_3_ (T4)	30.3 ± 0.004c	3.18 ± 0.001c	0.65 ± 0.001c
2 mg L^− 1^ Na_2_SeO_3_ (T5)	27.2 ± 0.004e	2.79 ± 0.001e	0.53 ± 0.001e
1.5 g L^− 1^ CaCl_2_ plus 1.5 mg L^− 1^ Na_2_SeO_3_ (T6)	21.4 ± 0.003f	2.10 ± 0.001f	0.42 ± 0.001f
**Different sampling times (vase day)**			
3rd	18.8 ± 0.003d	2.47 ± 0.001d	0.61 ± 0.001d
6th	26.7 ± 0.003c	2.59 ± 0.001c	0.64 ± 0.001c
9th	33.5 ± 0.003b	3.03 ± 0.001b	0.68 ± 0.001b
12th	42.8 ± 0.003a	3.82 ± 0.001a	0.71 ± 0.001a

Values represent means ± standard error *SE*. Not identical letters signify a substantial difference between the mean values as per Tukey test (*p* ≤ 0.01)

### Hydrogen peroxide

The H_2_O_2_ generation in control flowers quickly increased (Table 3), on the other hand, T3 and T4 treatments had the greatest impact on diminishing H_2_O_2_. It was evident that T6 generated the lowest substantial H_2_O_2_ levels (0.42 µmol^− 1^ g FW) when compared to all other treatments. Cut flowers’ H_2_O_2_ increased significantly and gradually over the course of 12 days of experiment, surpassing the first vase life day (3th day) by 16%. Cut flowers treated with CaCl_2_ and/or Na_2_SeO_3_ exhibited a decrease in the H_2_O_2_ levels at day 12 of vase life relative to comparable control flowers (Fig. 4C). Likewise, on day 12 of the experiment period, we noticed a decrease in H_2_O_2_ levels of 59% in T6-treated flowers over the corresponding control.

### Cell-wall-degrading-enzymes and catalase activities

**Fig. 5:**
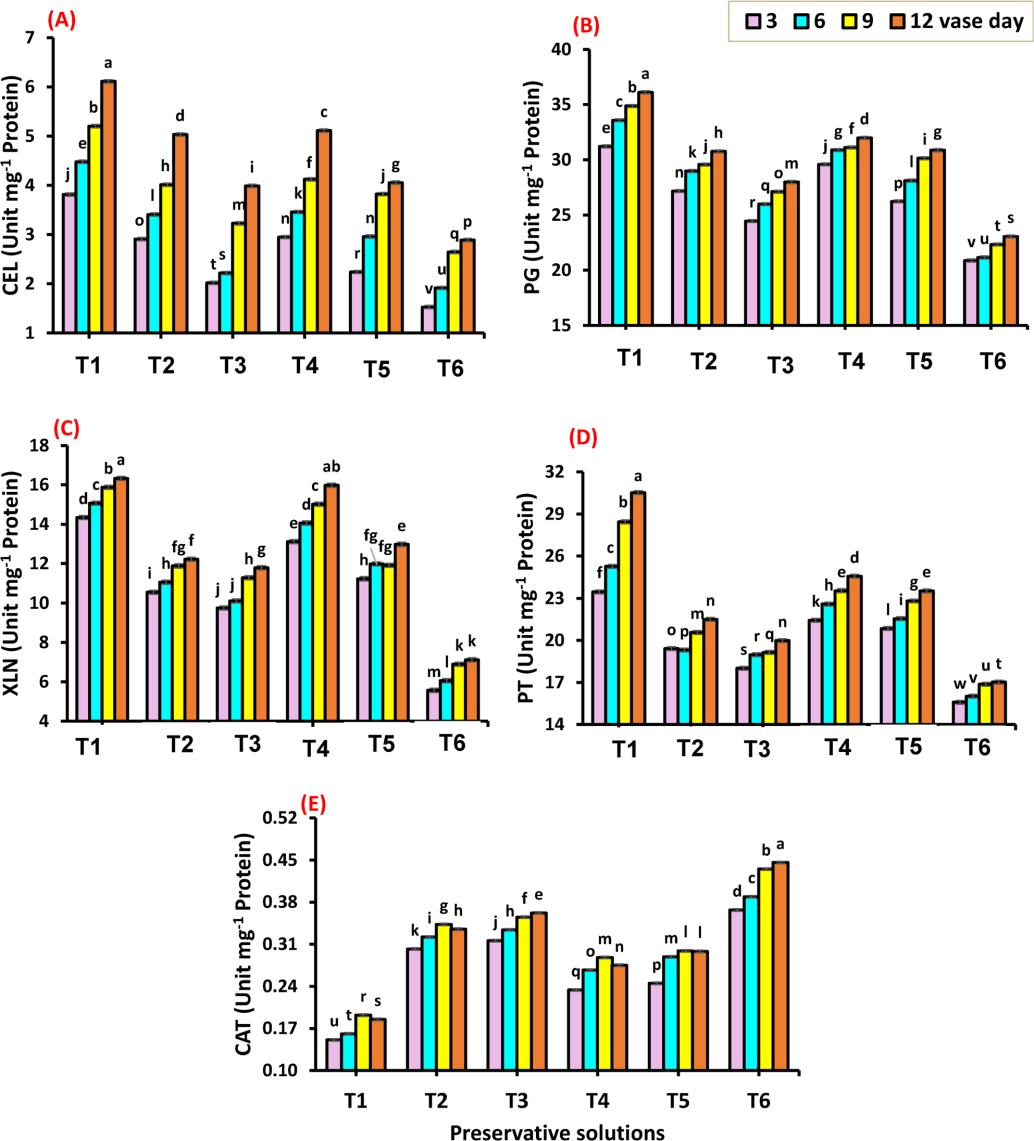
Cellulase (CEL; **A**), polygalacturonase (PG; **B**), xylanase (XLN; **C**), pectinase (PT; **D**), and catalase (CAT; **E**) activities in *Gerbera jamesonii* “Stanza” cut flowers affected by varied preservative solutions of distilled water as a control (T1), 1 g L^–1^ (T2) and 2 g L^–1^ (T3) of CaCl_2_, 1 mg L^–1^ (T4) and 2 mg L^–1^ (T5) of Na_2_SeO_3_, and combined 1.5 g L^–1^ CaCl_2_ with 1.5 mg L^–1^ Na_2_SeO_3_ (T6) over different sampling times. Bars represent mean values ± standard error (SE). Not identical letters signify a substantial difference between the mean values as per Tukey test (*p* ≤ 0.01)

In comparison to control flowers, the flowers treated with CaCl_2_ and/or Na_2_SeO_3_ decreased the activity of CEL, PG, XLN, and PT enzymes, and in contrast gradually increased the activity of CAT enzymes (Table 4). Furthermore, T6 treatment was the most significant effect on the enzyme’s activities, where CEL, PG, XLN, and PT values were 54, 35, 58 and 39-fold lower, and CAT value was 141-fold higher than of control flowers, respectively. There is a distinct trend of increasing CEL, PG, XLN and PT activities during the vase life period up to day 12, while the CAT activity increased slightly until day 9 of experiment before decreasing significantly on day 12 (Table 4). For the T6 solution, the lowest CEL, PG, XLN, and PT activities (2.89, 23.1, 7.11, and 17-unit mg^− 1^ protein, respectively), and the highest CAT activity (0.45-unit mg^− 1^ protein) on the last day of harvest (12th day) were noticed, which differed significantly from the control and CaCl_2_ or Na_2_SeO_3_ separately (Fig. 5A-E).

**Table 4:** Cellulase (CEL), polygalacturonase (PG), xylanase (XLN), pectinase (PT), and catalase (CAT) activities in gerbera cut flowers affected by varied preservative solutions and different sampling times (vase day)

Treatments	CEL	PG	XLN	PT	CAT
	Unit mg^− 1^ Protein				
**Preservative solutions**					
Distilled water (control; T1)	4.90 ± 0.001a	33.9 ± 0.000a	15.4 ± 0.03a	26.9 ± 0.002a	0.17 ± 0.000f
1 g L^− 1^ CaCl_2_ (T2)	3.84 ± 0.001c	29.1 ± 0.000c	11.4 ± 0.03d	20.2 ± 0.001d	0.33 ± 0.000c
2 g L^− 1^ CaCl_2_ (T3)	2.86 ± 0.001e	26.4 ± 0.001e	10.7 ± 0.04e	19.0 ± 0.002e	0.34 ± 0.000b
1 mg L^− 1^ Na_2_SeO_3_ (T4)	3.91 ± 0.001b	30.9 ± 0.001b	14.5 ± 0.03b	23.0 ± 0.002b	0.27 ± 0.000e
2 mg L^− 1^ Na_2_SeO_3_ (T5)	3.27 ± 0.001d	28.8 ± 0.001d	12.0 ± 0.03c	22.2 ± 0.002c	0.28 ± 0.000d
1.5 g L^− 1^ CaCl_2_ plus 1.5 mg L^− 1^ Na_2_SeO_3_ (T6)	2.24 ± 0.001f	21.9 ± 0.001f	6.41 ± 0.03f	16.4 ± 0.001f	0.41 ± 0.000a
**Different sampling times (vase day)**					
3rd	2.57 ± 0.001d	26.6 ± 0.001d	10.8 ± 0.001d	19.8 ± 0.001d	0.27 ± 0.000d
6th	3.07 ± 0.001c	28.1 ± 0.001c	11.4 ± 0.001c	20.6 ± 0.001c	0.29 ± 0.000c
9th	3.84 ± 0.001b	29.2 ± 0.001b	12.1 ± 0.001b	21.9 ± 0.001b	0.33 ± 0.000a
12th	4.53 ± 0.001a	30.1 ± 0.001a	12.7 ± 0.001a	22.9 ± 0.001a	0.31 ± 0.000b

Values represent means ± standard error (SE). Not identical letters signify a substantial difference between the mean

### Lignin content

**Fig. 6:**
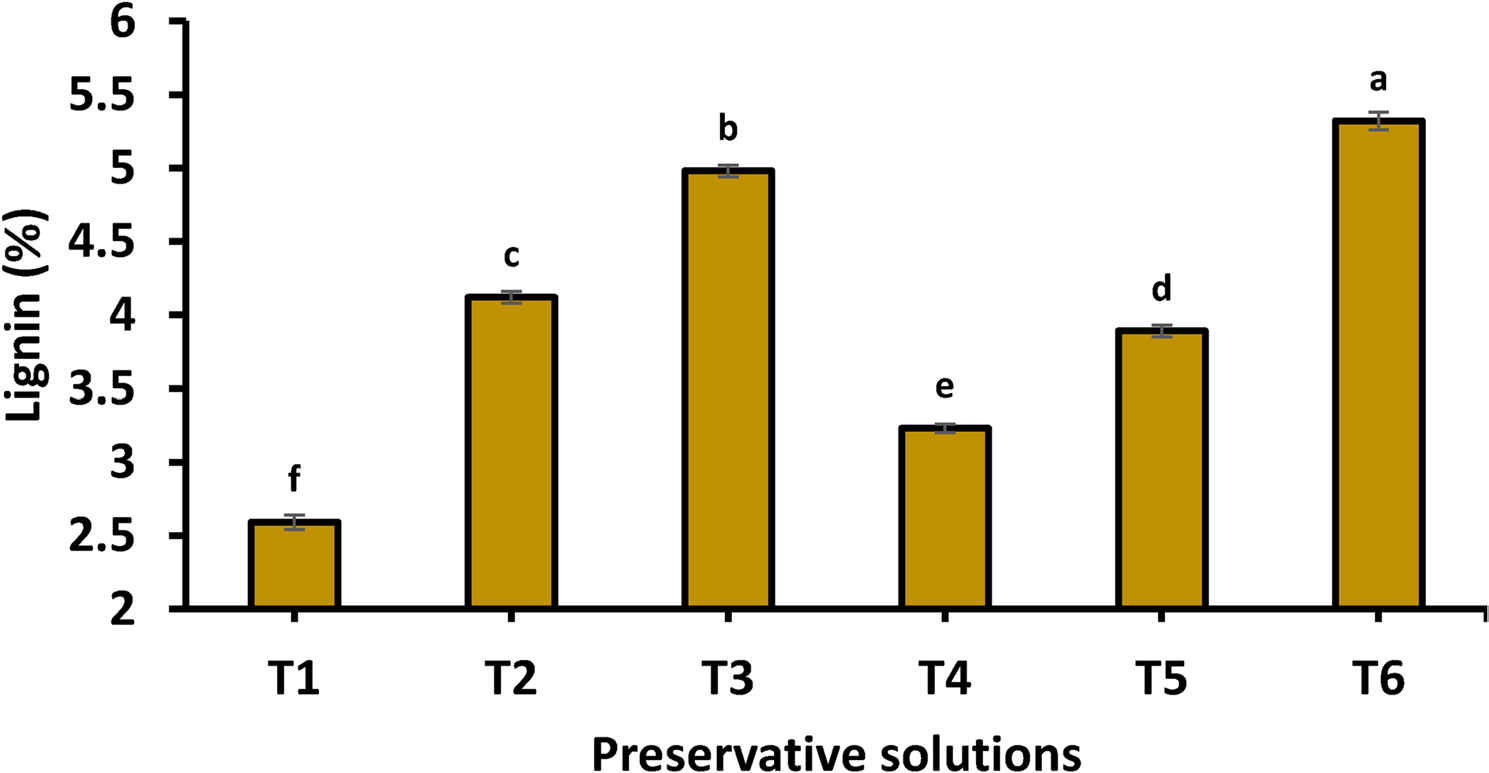
Lignin content in *Gerbera jamesonii* “Stanza” cut flowers affected by varied preservative solutions of distilled water as a control (T1), 1 g L^–1^ (T2) and 2 g L^–1^ (T3) of CaCl_2_, 1 mg L^–1^ (T4) and 2 mg L^–1^ (T5) of Na_2_SeO_3_, and combined 1.5 g L^–1^ CaCl_2_ with 1.5 mg L^–1^ Na_2_SeO_3_ (T6) over different sampling times. Bars represent mean values ± standard error (SE). Not identical letters signify a substantial difference between the mean values as per Tukey test (*p* ≤ 0.01)

The preservative solutions had an impact on lignin content as compared to the control (Fig. 6). In the cut section, T6-treated flowers had the highest lignin content (5.32%) while control flowers presented the lowest lignin content (2.59%) by an increase of 105%.

### Automatic Linear Modeling (ALM)

**Fig. 7:**
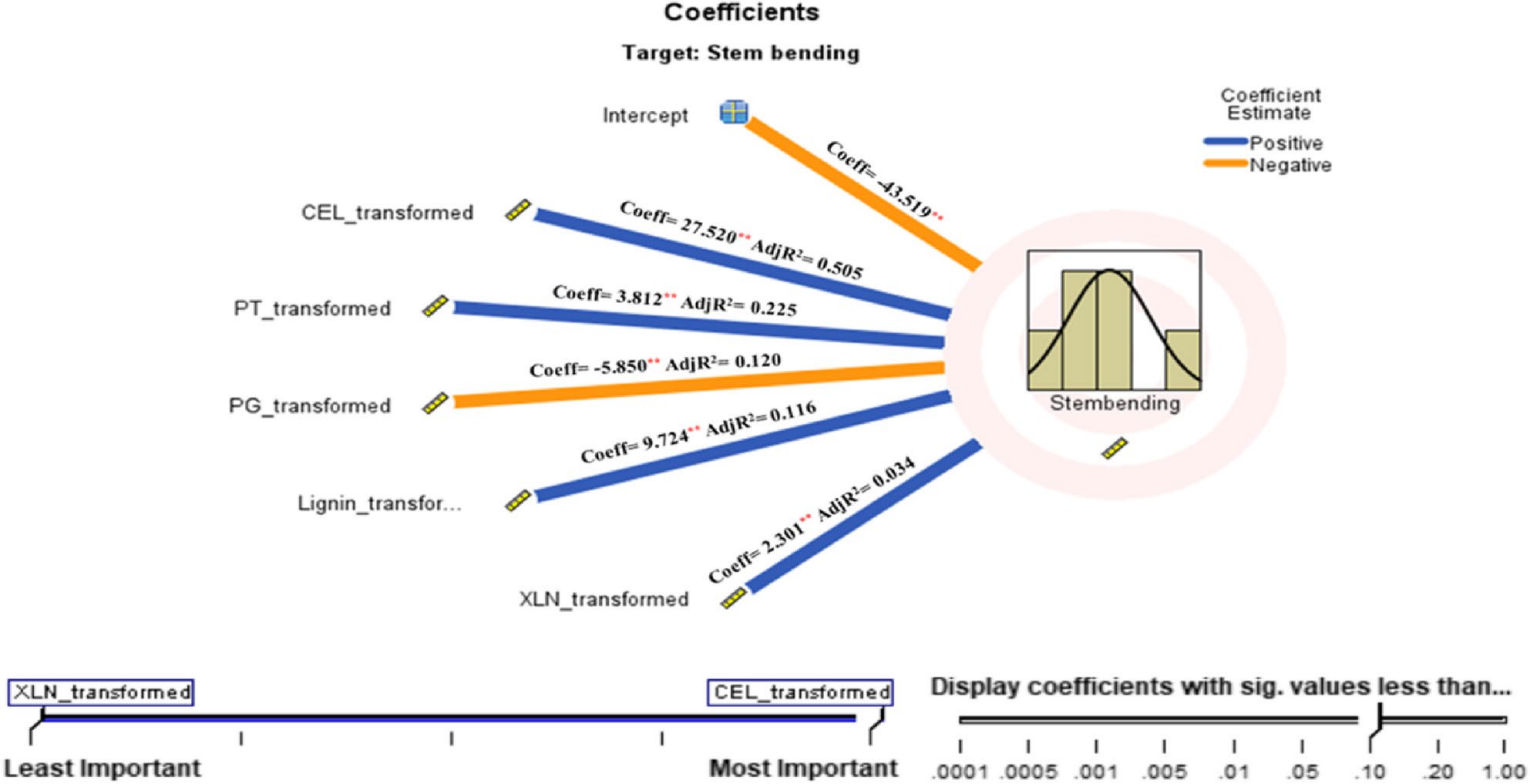
The automated linear modeling schematic for the influential qualities (e.g., CEL: cellulase, PT: pectinase, PG: polygalacturonase, lignin content, and XLN: xylanase) in stem bending of gerbera cut flowers by varied preservative solutions over different sampling times (vase life day). The acronyms Coeff and AdjR^2^ stand for coefficient-estimate, and adjusted-determination-coefficient, respectively. ** denotes significance levels at *p* ˂ 0.01

ALM results showed that forward-stepwise-regression (FSR) explains the relation between stem bending as a target characteristic and its explanatory contribution (predictors) characteristics under study (Fig. 7). Adjusted R^2^ is 0.999 for the outputted-FSR-model, indicates that 99.9% of the variations in stem bending are interpreted by variations in CEL, PT, PG, lignin, and XLN (stem bending = − 43.519^**^ + 27.520^**^ CEL + 3.812^**^ PT − 5.850^**^ PG + 9.724^**^ lignin + 2.301^**^ XLN). These findings highlight the critical roles of cell-wall-degrading-enzymes, and lignin content in controlling stem bending in gerbera cut-flowers under study.

## Discussion

Flower-senescence is a crucial physiological procedure that shortens the vase-life of cut-flowers and influences their overall quality [40]. Stem bending, cell death, lipid, protein depletion, water loss, and nucleic acid degradation, as well as, a reduction in ornamental value are typical consequences of cut-flower-senescence [41]. Generally, flower-senescence cannot be prevented, but it can be controlled by using a number of postharvest strategies. Park and Kim [42] indicate that stem bending shortens the vase life of *Gerbera jamesonii*. Stem bending has been linked to a number of factors, including low content of lignin, a lack of mechanical support, xylem obstruction [8], water deficit, increased ROS [43], and enzymes activities that degrading cell walls, as well as a decrease in non-enzymatic substances (phenols and anthocyanins), and antioxidant defense enzyme (CAT).

Contrary to the controls, CaCl_2_ and/or Na_2_SeO_3_ significantly prolonged the vase life of cut gerbera ‘Stanza’ flowers (Fig. 1A). The combined 1.5 g L^− 1^ CaCl_2_ with 1.5 mg L^− 1^ Na_2_SeO_3_ treatment prolonged the vase life of gerbera cut flowers by 107% as compared with control flowers. This improvement could be due to the combined direct effects of downregulating-ethylene-biosynthesis [44], and indirectly effects by lowering flower senescence-related processes, like preserving water balance, decreasing oxidative stress and increasing the antioxidant enzyme activities, as well as, lowering respiration rate, carbohydrate degradation, petal wilting during the vase period [45]. The results presented in this study are similar to those reported by Zhou et al. [46] on carnation, Ehsanimehr et al. [47] on rose, and Mohammadi et al. [43] on gerbera. The current findings demonstrate that, CaCl_2_ and/or Na_2_SeO_3_ considerably enhanced TSU during the vase life of gerbera flowers (Fig. 1B). This is due to the role of Ca in forming a compound in the middle plate between the membranous-cell-wall and poly-galacturonic acid to strengthen the cell-membranes, which leads to the accumulation of pectin in the xylem-cellular-walls, which facilitates the easy movement of water from the cut stems [48]. Furthermore, increasing TSU may be due to the Se’s antibacterial properties that prevent xylem occlusion or its beneficial role in locking the pores that control evapotranspiration and the petals’ ability to retain water [49]. Comparable outcomes have also been seen in carnation [46], rose [47], and gerbera [43]. Preserving RFW throughout the vase duration in order to prolong the vase life is crucial. Although a gradual decline in this factor over the course of post-harvest life is expected, a smaller weight loss is a reliable indicator of the greater impact of the treatments utilized. Compared to control test solutions, the RFW of cut gerbera flowers was impacted by the CaCl_2_ and/or Na_2_SeO_3_ treatments. Until the 9th day, the treatment combining 1.5 g L^− 1^ CaCl_2_ and 1.5 mg L^− 1^ Na_2_SeO_3_ had the largest value of RFW (Fig. 2A). Gururani et al. [50] stated that the increase in respiration rate is the main reason in decreasing the fresh weight. Therefore, the increase in RFW may be due to the combination effect of reducing respiration and transpiration ratios caused by closing stomata and/or increasing water intake by cut flowers [51]. These results align with previous studies on gerbera [52, 53) and rose [45]. In parallel with supplying gerbera cut flowers with CaCl_2_ and/or Na_2_SeO_3_, and extending the experiment period to 12 days, an increase in the diameter of the gerbera flowers was observed (Fig. 2B). The present results were in line with Xia et al. [52] on gerbera and Wang et al. [45] on rose. This accomplishment may be the result of these substances’ impact on physiological processes in the ligules, which increase solution uptake in cut flowers by blocking xylem blockage and reducing the activity and effect of certain enzymes that synthesize lignin [53].

Stem bending progressed with an increasing vase life period and reached its maximum on the last day of sampling (Table 1; Fig. 2C). However, supplying flowers mix 1.5 g L^− 1^ CaCl_2_ with 1.5 mg L^− 1^ Na_2_SeO_3_ significantly reduced stem bending at a pedicel curvature. According to García-González et al. [54], stem bending in gerbera is mostly caused by turgor loss in the mid-stem region, which is connected to the central cavity that begins 5 cm above the basal region and ends 10 cm below the flower head. Stem bending results from firmness loss brought on by decreased turgidity [55]; however, in our experiment, we obtained a decrease in the pedicel curvature following Ca and Se solutions. Their effect could be caused by the linking of pectin molecules in the middle lamella, which would increase the stiffness of the cell wall [56, 57]. Furthermore, Ca greatly increases the structural rigidity of plant cell walls, which serve as the primary mechanical support system for the whole plant [58]. To date, no studies have been done on the effects of Se on stem bending and senescence of gerbera-cut-flowers. However, this may be due to the role of Se as an anti-bacterial agent, which results in preventing bacterial obstruction in the xylem [21], and thus reducing stem bending. Additionally, the reason for the reduction in pedicel curvature may be what we have verified in our study that Ca and Se play a role in reducing cell wall-degrading and activating-oxidative enzymes, as well as increasing the lignin content at the end of stem section (Table 4, and Fig. 6).

CaCl_2_ and/or Na_2_SeO_3_ treatments considerably increased the MSI level, which was more effective than the control treatment, during the vase life period (Fig. 3A). The apparent stability of membranes in Ca and Se solutions may be accounted for *via* multiple physiological mechanisms. The first possibility is supplying the flowers with increased their bio-availability and sustained-release, extending the antioxidant benefits of Se [46]. Second, the combined action of Ca’s creation of a protective barrier and Se’s scavenging of free radicals may prevent lipid peroxidation and maintain membrane phospholipids stability [20]. The current findings concurred with those of Mohammadi et al. [43] found that CaCl_2_ decreased lipid peroxidation and increased MSI in gerbera flower. These discoveries undoubtedly contributed to delaying senescence, thus extending vase life and mitigating stem bending in cut gerbera flowers. The decrease in TAC seen in control flowers until the last day of sampling (12th day, Table 2) is consistent with the usual degradation patterns linked to petal-senescence. Current results were similar to García-González et al. [54] on gerbera and Mobaraki et al. [59] on roses. TAC, actuality vacuolar pigments, is prone to deterioration as cell integrity declines throughout senescence [60]. The superior performance of Ca and Se solutions in enhancing TAC until the end of the vase period can be attributed to a variety of factors. This may be due to Ca’s role in preserving cellular and vacuolar-integrity, maintaining cellular-membranes and protecting TAC from degradation [61]. Furthermore, Se’s powerful antioxidant role may have significantly improved antioxidant defense, thus safeguarding TAC from oxidative degradation [62]. Likewise, the interaction of Ca’s physiological actions with Se’s defensive properties may have produced a favorable environment (optimal pH) that supported the maintenance of TAC and synthesis [63]. A significant increase in TPC of gerbera petals, particularly on the 9th day of the vase period was noticed (Table 2). However, the flowers treated with 1.5 g L^− 1^ CaCl_2_ + 1.5 mg L^− 1^ Na_2_SeO_3_ had the highest increase in TPC by day 12, surpassing the controls by 253.6% (Fig. 3C). Our findings were in line with recent studies that found greater TPC *via* Ca solutions over longer storage times for cut roses [47], and cut gerbera [43]. The divergent reactions of TPC to the solutions demonstrate how intricate the dynamics of secondary metabolites are during petal senescence [64]. This pattern may be explained as a stress response strategy in which the plant produces phenolics to protect itself against oxidative stress linked to senescence [65]. This result may be due to the role of Ca in activating some enzymes such as phenylalanine ammonia lyase (modulate several physiological-processes), which leads to an increase in phenols synthesis [66]. Also, Se can trigger defense mechanisms in plants, including the synthesis of secondary metabolites [46]. Likewise, Ca and Se together might have enhanced TPC biosynthesis in a synergistic way.

Under a range of biological and abiotic stresses, plants typically generate large amounts of ROS in their cells [67]. These ROS have the ability to alter the cell’s dynamic balance and interfere with plant physiological functions [68]. Plants can increase their resilience to both abiotic and biotic stresses by utilizing their antioxidant system and secondary metabolism. The changes observed in IL rate, MDA content, H_2_O_2_ level, CEL, PG, XLN, and PT values as well as, CAT activity in cut-gerbera-flowers provided a thorough illustration of the dynamics of oxidative-stress and antioxidant-reactions. These results point to overarching discussion of the fundamental physiological processes and the ensuing repercussions [69] for cut-gerbera-flower longevity and stem bending. In our study, up to the last day of sampling, we noticed a decrease in Il rate, MDA content, H_2_O_2_ levels (Table 3; Fig. 4), and postponed cell-wall-degrading-enzymes by controlling CEL, PG, XLN, and PT, as well as promoting the activity of CAT enzyme in mixture (1.5 g L^− 1^ CaCl_2_ + 1.5 mg L^− 1^ Na_2_SeO_3_) treated cut flowers. Ca and Se solutions significantly reduced IL. The results were consistent with Mobaraki et al. [59], also reported that Ca improves membrane integrity, leading to the removal of oxidative-tensions and reducing IL by increasing ROS scavenging activity [61]. Zhou et al. [46] excessive ROS formation is a feature of leaf senescence, and the steady increase in H_2_O_2_ level and MDA content indicated that the cell membrane’s integrity deteriorates as the senescence process progresses. The Ca and Se solutions, however, dramatically decreased the occurrence; this suggested that the biofortification of Ca and Se and their role in enhancing the synthesis of phenolics, as secondary metabolites [61]. The findings of this investigation are comparable to those published by Mohammadi et al. [43] on gerbera and SeyedHajizadeh et al. [17] on black magic roses.

We noticed that all treatments had higher cell wall enzymes activities. The flower’s stem showed a rise in CEL, PG, XLN, and PT (Table 4; Fig. 5), and these enzymes’ activities rose starting on day 3. The 1.5 g L^− 1^ CaCl_2_ + 1.5 mg L^− 1^ Na_2_SeO_3_ solution significantly inhibited these increases and prevented the stem bent degree from increasing as well. Therefore, an increase in this enzymes activity in the bending region may be partially responsible for stem bending. Additionally, an increase in CAT enzyme activity, especially in flowers treated mixed solution demonstrate the upregulation of enzymatic antioxidant protection. This impact may result from Ca’s fundamental function of binding with the free carboxyl-groups located at the galacturonic acids of the middle lamella’s cell wall pectins [70], making cell-walls more robust and better able to withstand enzyme breakdown [71]. Moreover, Se’s function in plant metabolism and physiology, as well as its capacity to support plant antioxidant control [17]. According to reports, Se contributes to the organization and signaling of gene expression during the senescence of *Arabidopsis* leaves [72]. The results of this study are similar to those that were observed by Zhou et al. [46] on carnation, Ehsanimehr et al. [47] on rose, and Mohammadi et al. [43] on gerbera. A previous study found that the lignin production pathway is a crucial part of the phenylalanine metabolic system and a secondary metabolic step that is vital to plant defense [23]. Plant-secondary-cell-walls require lignin (called a phenolic polymer) [73]. It can strengthen plants mechanically, provide them with physical and chemical protection, and increase their resistance to diseases [74]. Low lignin content weakens the vascular tissue and stem’s hardness, which reduces the flower’s overall mechanical resistance and prevents water transmission [75], causing stem bending quickly. Ca or Se’s ability to maintain lignin content (Fig. 6) may be linked to its ability to suppress the synthesis of ethylene and increasing the activity of the antioxidant enzyme CAT [76]. Furthermore, Se may also play a role in the synthesis of phenylalanine-ammonia-lyase-enzyme [77], that catalyzes the initial stage of the phenylpropanoid-metabolism and is in charge of the synthesis of numerous secondary chemicals linked to defense [78, 79] such as lignin.

**Fig. 8:**
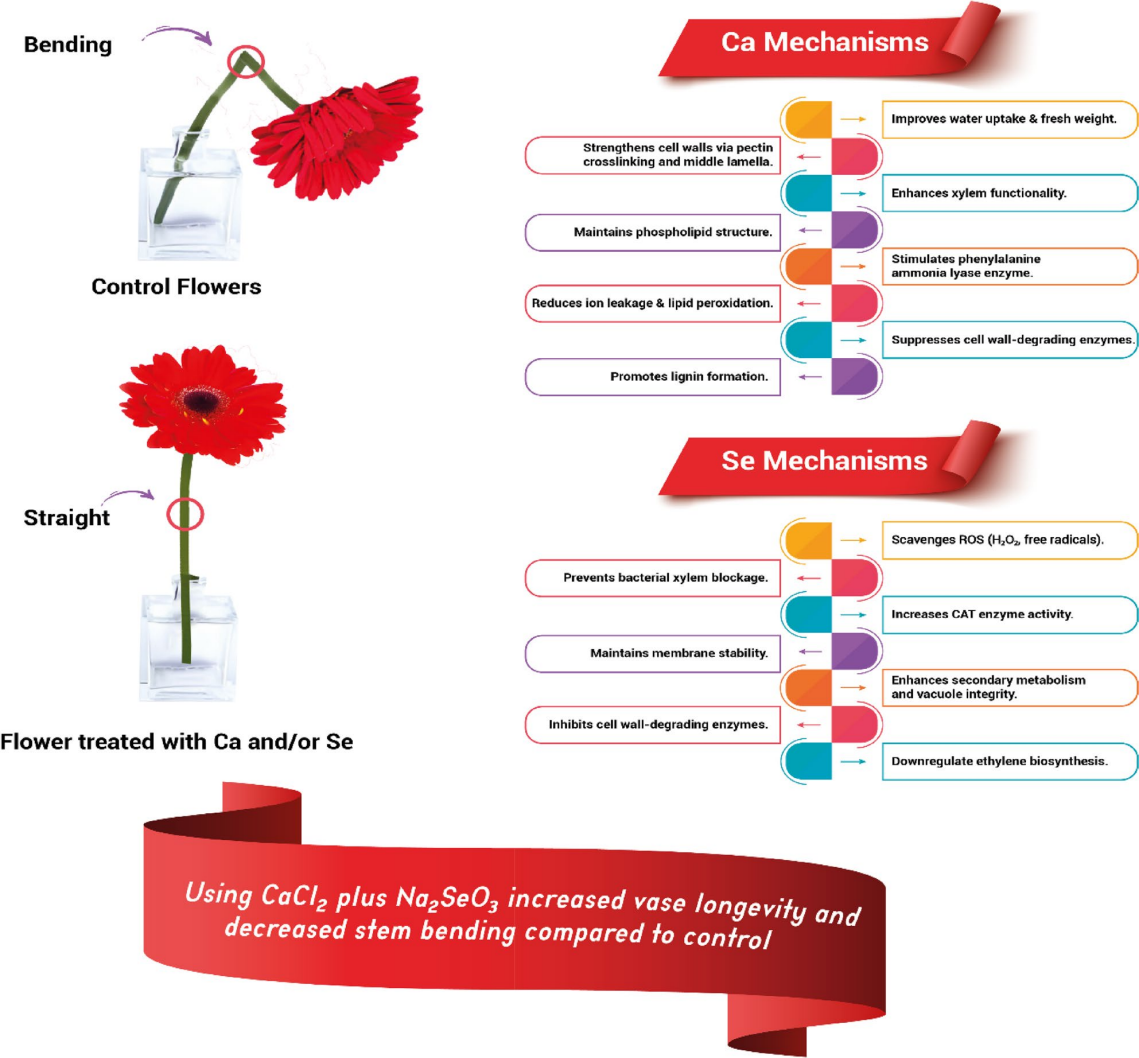
Summary graphical of the suggested mechanisms for the effect of CaCl2 and/or Na2SeO3 on vase life and stem bending in gerbera cut flowers

In this study, we propose a new model for the beneficial metabolic interaction between Ca and Se, and its impact on the postharvest life of bend-susceptible gerbera flowers. A key aspect of our findings is that the functional application of these treatments aligns strongly with established scientific as well as regulatory standards for plant biostimulants [80]. Even though Ca is a nutrient, its primary function in our study was structural, since it enhanced cell wall integrity to reduce the abiotic stress caused by stem bending. Se, a non-essential element, acts directly as a biostimulant by activating the plant’s internal antioxidant systems. Commercially, this approach is highly viable; CaCl_2_ and Na_2_SeO_3_ are cost-effective, and easily integrated into existing postharvest protocols. Therefore, their demonstrated ability to significantly extend vase life clear financial benefits to the floriculture industry by boosting market value and reducing waste (Fig. 8).

## Conclusion

This is the first study confirming that Ca and Se are capable of maintaining the post-harvest properties of cut-gerbera-flowers in terms of prolonging their vase life and alleviating the stem bending. These results suggest that scape bending of cut-gerbera-flowers may be linked to the lack of mechanical support on the stem and regulation of ethylene signaling. Ca’s role in the structural rigidity of plant cell walls and lignin formation, plus Se’s powerful antioxidant role led to a reduction in cell-wall-degrading-enzymes, and an increase in synthesis of secondary metabolites. Hence, we propose that Ca and Se represent a new strategy as a preservation solution to prolong the vase life of cut-gerbera-flowers and reduce stem bending.

## Data Availability

Data availability: All data generated or analysed during this study are included in this published article.

## Abbreviations

CaCl_2_: Calcium chloride
Na_2_SeO_3_: Sodium selenite
SB: Stem Bending
CEL: Cellulase
PG: Polygalacturonase
XYL: Xylanase
PT: Pectinase
CAT: Catalase
IL: Ion leakage
MDA: Malondialdehyde
H_2_O_2_: Hydrogen peroxide
RFW: Relative Fresh Weight
TSU: Total Solution Uptake
MSI: Membrane Stability Index
TAC: Total Anthocyanin Content
TPC: Total Phenols Compounds
ALM: Automatic Linear Modeling
FSR: Forward-stepwise-regression
